# Simulating Cardiac Electrophysiology Using Unstructured All-Hexahedra Spectral Elements

**DOI:** 10.1155/2015/473279

**Published:** 2015-10-25

**Authors:** Gianmauro Cuccuru, Giorgio Fotia, Fabio Maggio, James Southern

**Affiliations:** ^1^CRS4, Loc. Pixina Manna, Edificio 1, 09010 Pula, Italy; ^2^Fujitsu Laboratories of Europe, Hayes Park Central, Hayes End Road, Hayes, Middlesex UB4 8FE, UK

## Abstract

We discuss the application of the spectral element method to the monodomain and bidomain equations describing propagation of cardiac action potential. Models of cardiac electrophysiology consist of a system of partial differential equations coupled with a system of ordinary differential equations representing cell membrane dynamics. The solution of these equations requires solving multiple length scales due to the ratio of advection to diffusion that varies among the different equations. High order approximation of spectral elements provides greater flexibility in resolving multiple length scales. Furthermore, spectral elements are extremely efficient to model propagation phenomena on complex shapes using fewer degrees of freedom than its finite element equivalent (for the same level of accuracy). We illustrate a fully unstructured all-hexahedra approach implementation of the method and we apply it to the solution of full 3D monodomain and bidomain test cases. We discuss some key elements of the proposed approach on some selected benchmarks and on an anatomically based whole heart human computational model.

## 1. Introduction

Mechanistic models of heart physiology are emerging as an important paradigm able to complement the insight and exploit the information provided by genetics and biological experiments. Very recent years have seen the first attempts to define models for the numerical simulation of the individual response of real cardiology patients. For instance, in [1] the clinical study of a 65-year-old male with ischemic cardiomyopathy was addressed by means of a workflow consisting of detailed anatomic reconstruction and effective modelling of electrical activity, biomechanics, and hemodynamics. The modelling steps are challenging because they require performing (i) multiple simulations to estimate parameters and test outcomes of different treatment options and (ii) many large scale computer analyses in a clinically useful time-frame. These are strong motivations in favour of effective, anatomy-compliant high performance computer methods.

In particular, current software to handle the forward problem of the electrocardiology, that is, modelling the electrical activity of the heart, largely fails to deliver the performance needed for clinical applications. Typical discretization techniques applied to this problem have an average nodal spacing of 0.2 mm or less, resulting in approximately 30 million grid points for an average-size human heart. This possibly gives rise to large scale computational problems which are difficult to solve in real-time. The most common approach relies on the use of large high performance computer systems featuring 10^3^ to 10^4^ cores [2, 3], a choice often beyond the possibility of typical end-users. Several strategies have been suggested for facing this hindrance, including simplifying/optimizing the cardiac cell models [4], code optimization for run on next-generation hardware devices [5], and adoption of more effective solutions for the approximations of the differential equations at hand. This work is based on the latter approach.

## 2. Related Work

The bidomain equations [6] are a commonly used model for the propagation of depolarization wavefronts across cardiac tissue. They are often solved numerically using finite elements (FE), finite volumes or finite differences, and a variety of algorithms based on these techniques have been proposed [7–14]. However, each of these methods suffers from a well-known limit affecting the numerical simulation of propagation phenomena: to prevent the onset of nonphysical, spurious effects commonly referred to as numerical dispersion it is necessary to properly fix the accuracy of the method. In cardiac simulation this has most commonly been achieved by using grid refinement, that is, choosing a node spacing that is sufficiently fine that numerical dispersion is not a problem.

Alternatively, one can improve the quality of numerical simulations by using a relatively coarse mesh, increasing the polynomial degree of the basis functions used in the numerical scheme. This is the case of high order methods, examples of which are the *p*-version of the finite element method and the spectral element method. High order methods possess many advantages over standard techniques, such as considerably higher convergence rates, and, in our experience, the possibility of using elements with large aspect ratios without significant deterioration in accuracy. Designed to combine the good accuracy properties of pseudospectral techniques such as Legendre or Chebyshev methods with the geometrical flexibility of classical low order FE methods, spectral element methods were first introduced in 1984 [15], combining spectral methods with domain decomposition approaches. During the last decades, spectral elements have firmly established as an effective tool to treat diverse compute-intensive physical problems on complex geometries in fluid dynamics, continuum mechanics, geophysics, and electromagnetics; see, for example, [16] and references therein.

As for our knowledge, few attempts have been done to exploit spectral elements in electrocardiology. An interesting idea is provided by the so-called spectral smoothed boundary method [17], which tries to merge the excellent convergence property of Fourier-type spectral methods (which, on the other hand, are not able to accommodate irregular geometries) with the phase-field method, an embedding approach capable of dealing with complex domains when no-flux boundary conditions are present. In [18], high order spectral/hp elements on curvilinear triangles are used to model electrical propagation in the heart surface, addressing the monodomain problem and including anisotropic heterogeneous propagation in presence of fibre orientation. In [19], which in the authors' words “should be seen as a proof-of-concept,” high order finite elements based on triangles have been applied to the solution of the monodomain problem in 1D and 2D reference geometries: furthermore, authors provide rigorous analysis and numerical experiments on theoretical error convergence rates and suggest the possibility to extend their method to spatial adaptivity.

When solving numerically the electrocardiology problem, extremely high accuracy is not a valuable goal. To be realistic, none of the numerous parameters involved in the mono-/bidomain model is known with a precision higher than 10%. On a logical ground, there is no use in solving with outstanding accuracy a problem based on a model which is only a partial approximation of a real patient cardiovascular system. In this frame, the rationale behind the use of spectral elements is the possibility to achieve an a priori fixed, reasonable accuracy using less degrees of freedom (DOF) with respect to low order methods. Matrices resulting from space discretization are more dense but have a smaller overall number of nonzero entries: this is important for decreasing both memory footprints and execution times. On the other hand, a reduced number of DOFs mean smaller ODE systems describing cell models, possibly shrinking the CPU-time for the* in silico* analysis.

Based on our experience in several fields of engineering, we propose to use straightforward, fully unstructured all-hexahedra spectral elements (SEM in the sequel). Differently from high order methods based on triangular elements, the SEM rely on a plain, standard, consolidated, widely accepted mathematical formulation based on tensor product, which brings tangible advantages in practical algorithm implementation. Comparison with triangular spectral elements is a difficult task as the latter came up in many different algorithm implementations and most of the works on theory and numerical experiments concern the 2D case. For instance, in [20] Koornwinder-Dubiner polynomials and Fekete points were chosen as orthogonal basis and approximation points, respectively. This choice brings differentiation matrices greater in size with respect to their SEM counterpart, thus raising the computational effort for matrix-vector products and, consequently, the overall CPU-time. Remarkably, numerical experiments suggest that the condition number of triangular spectral matrices is worse than the SEM ones, with clear advantage for the latter in terms of convergence of iterative methods and error propagation. Furthermore, preconditioners for SEM have been long studied and developed; see, for instance [21] and references therein. Apart from these good mathematical properties, simplicity of SEM results in easy to implement, readable computer codes enjoying good portability to high performance platforms and new hardware devices like GPGPUs (as, for instance, there is no need for complex data-structures). Also, since they share a common philosophy with traditional finite elements, they could benefit from several existing and well-consolidated tools (pre- and postprocessing routines, mesh partitioning, iterative solvers, preconditioners, etc.).

The other side of the picture is that all-hexahedral grids are more difficult to generate than simplicial tetrahedral grids. While automatic (or semiautomatic) hex-only mesh generation for complex geometries is an active subject of research, generation of an all hexahedral mesh for applications in electrophysiology requires nowadays more effort than hybrid or all-tet grids. This is particularly true for those studies where anatomical properties should be resolved at a very fine anatomical detail. Clearly, the extent to which a study requires representation of structural detail is determined by the particular application and there might be some cases where this is not a serious limitation. Finally, the spectral element method implementation we propose is restricted to 8-node hexahedra. Extension to (curved) isoparametric elements is currently being addresses and will allow mapping curved boundaries of irregularly shaped anatomical details and represent geometries more closely.

## 3. Materials and Methods

### 3.1. The Bidomain Model

For a 3D domain *Ω* the bidomain equations in parabolic-parabolic form read [22]

(1a) χC∂V∂t−∇·σi∇ϕi=−χIion+Istim,i,

(1b) −χC∂V∂t−∇·σe∇ϕe=χIion+Istim,e,

(1c) ∂w∂t−RV,w=0,

(1d) ∂c∂t−SV,w,c=0,

where *ϕ*_*i*_(**x**, *t*) and *ϕ*_*e*_(**x**, *t*) are the intracellular and extracellular potentials, *V*(**x**, *t*) = *ϕ*_*i*_ − *ϕ*_*e*_ is the transmembrane potential, *C* is the membrane capacitance per unit area, *σ*_*i*_ and *σ*_*e*_ are the intracellular and extracellular conductivity tensors modeling the anisotropy of the cardiac tissue, and *χ* is the cell surface area to volume ratio. Here, **w** and **c** are arrays of ionic gating variables and ionic concentrations, respectively [22], and *I*_stim,*i*_ and *I*_stim,*e*_ are externally applied intracellular and extracellular stimulus currents. Suitable boundary conditions on either *ϕ*_*i*,*e*_ or the current flux **n** ^*T*^ *σ*_*i*,*e*_∇*ϕ*_*i*,*e*_, and initial conditions for *V*, **w**, and **c** are set. Functions **R**, **S** and the transmembrane current *I*_ion_ ≡ *I*_ion_(**w**, **c**, *V*) are determined by parameters and data fitting and represent an electrophysiological cell model. Many of such cells models of variable complexity exist; see, for example, [23] for a recent review. In this work we model the ionic current using the phase-I Luo-Rudy (LR1) cell model [24]. However, the derivation of the SEM formulation of the bidomain model follows analogously for any ordinary differential equation (ODE) based cell model. The LR1 model relies on six gating variables {*w*_1_,…, *w*_6_} along with the intracellular calcium concentration *c*_1_. The compatibility condition

(2) $$\begin{array}{c} \int_{\Omega }\left(\;I_{\text{stim},i}+I_{\text{stim},e}\;\right)d\Omega =0 \end{array}$$

on *I*_stim,*i*_ and *I*_stim,*e*_ should hold for the system to be solvable. Note that the transmembrane potential *V* is uniquely determined, while the intra- and extracellular potentials *ϕ*_*i*_ and *ϕ*_*e*_ are determined up to the same additive time-dependent constant, whose value is usually obtained by imposing a normalization condition on *ϕ*_*e*_, for example, zero average on *Ω*.

We remark that a form alternative to (1a)-(1b), the parabolic-elliptic formulation of the bidomain equations, exists, with unknowns (*ϕ*_*e*_, *V*). Such formulation is quite popular, even because it allows dealing with the parabolic and elliptic blocks at different steps by Gauss-Seidel method. Nevertheless, (i) recent studies show that decoupling the bidomain equations can be less efficient than solving them as a global system (for the same level of accuracy [25]), and (ii) evidence exists that formulation (*ϕ*_*e*_, *ϕ*_*i*_) provides a significantly more efficient numerical framework [26]. Our SEM implementation addresses the coupled parabolic-parabolic problem, even because, being the SEM mass matrix diagonal, this allows a significant save in memory allocation and, quite likely, a reduced computational effort. This is because the number of nonzero entries of the global matrix (hence the cost for sparse matrix-vector products) is greatly reduced with respect to the parabolic-elliptic formulation.

### 3.2. Variational Formulation of the Bidomain Model

The variational form of the bidomain equations is as follows: given *I*_ion_ and *I*_stim,*i*,*e*_ fulfilling the compatibility condition (2), for all *t* > 0, find *ϕ*_*i*,*e*_ ∈ *H* ^1^(*Ω*) × *H* ^1^(*Ω*)/{(*c*, *c*) : *c* ∈ *ℝ*} satisfying given initial and boundary conditions such that

(3a) χC∂V∂t,ψi+aiϕi,ψi+χIion,ψi=Istim,i,ψi,

(3b) −χC∂V∂t,ψe+aeϕe,ψe−χIion,ψe=Istim,e,ψe

for all admissible test functions *ψ*_*i*,*e*_ from a suitable test space, where *a*_*i*,*e*_(*u*, *v*) = ∫_*Ω*_∇*u* ^*T*^ *σ*_*i*,*e*_∇*v* *dΩ*. See [27] for a mathematical analysis of the bidomain model. Existence and uniqueness for a solution of the bidomain problem for a wide class of cell models, including LR1, are discussed in [28].

### 3.3. Spatial Discretization

Let *𝒯*_*h*_ be a decomposition of the model volume *Ω* into a family of nonoverlapping hexahedra *Ω*_*k*_ with typical size *h*. Each element *Ω*_*k*_ is obtained by a transformation *F*_*k*_ from a reference (or parent) element $\hat{\Omega }={\left[-1,1\right]}^{3}$. On the reference element, we consider the space *ℚ*_*p*_ of polynomial functions with degree less than or equal to *p* in each variable *x*_*i*_, *i* = 1,2, 3. Then, for each *Ω*_*k*_ let

(4) $$\begin{array}{c} U_{p,k}\left(\;\Omega_{k}\;\right)=\left\{\;u=\hat{u}\circ F_{k}^{-1},\text{ for some }\hat{u}\in Q_{p}\left(\;\hat{\Omega }\;\right)\;\right\} \end{array}$$

be the space of the functions that are images through *F*_*k*_ of polynomials functions $\hat{u}\in {\hat{\mathbb{Q}}}_{p}$ in $\hat{\Omega }$. Hence, $u(x)=\hat{u}(F_{k}^{-1}(x))$ for all **x** in *Ω*_*k*_. Finally, we define the spectral element space as

(5) Xh,pΩ≔u∈C0Ω¯:uΩk∈Up,kΩk,  ∀Ωk∈Th.

In our implementation *F*_*k*_ ∈ *ℚ*_1_; thus, when *p* > 1 the mapping is subparametric, meaning that its degree is lower than the spectral degree. This choice is essentially motivated by practical considerations: mesh generators produce 8-point hexahedra, while *p*-order hexahedra need (*p* + 1)^3^ total points (and produce different grids for analyses with different spectral degree). The subparametric mapping is known to enjoy good mathematical properties (see, for instance, [29]). Nevertheless, if high order hexahedra are desirable (for instance, when dealing with domains with curved boundary which can be described in terms of geometrical primitives), they can be incorporated in the spectral element frame with small additional effort.

### 3.4. Construction of SEM Basis Functions

First, the Gauss-Lobatto (LGL) points in the reference element $\hat{\Omega }$ are obtained via tensor product of the one-dimensional LGL nodes in [−1, +1]. The full spectral grid {**a**_*p*_}_*p*=1_^*N*^ is then built mapping the LGL nodes over the hexahedra and eliminating duplicated points: as for finite elements, a global numbering is associated with the *N* grid points. In $\hat{\Omega }$ we consider the (*p* + 1)^3^ Lagrange polynomials of degree *p* corresponding to the LGL nodes and, in *Ω*_*k*_, the elemental basis functions for *U*_*p*,*k*_ obtained by mapping such nodal basis functions according to *F*_*k*_. Finally, a basis for the whole space *X*_*h*,*p*_ is obtained as a patchwork of these elemental functions on each element *Ω*_*k*_. More precisely, we choose as a basis for *X*_*h*,*p*_ the set {*N*_*p*_(**x**)}_*p*=1_^*N*^, that is, the transformation of polynomials of order *p* from $\hat{\Omega }$ to each *Ω*_*k*_, such that

(6) $$\begin{array}{c} N_{p}\in X_{h,p},\;N_{p}\left(a_{q}\right)=\delta_{pq}, \end{array}$$

where *δ*_*pq*_ is the Kronecker delta. It is easy to see that the restriction of such spectral shape functions to *Ω*_*k*_ either coincides with a Lagrange polynomial or vanishes. It is easily verified that the above choice of nodal basis functions assures that the corresponding global bases enjoy as much localization as possible.

### 3.5. SEM Formulation

Taking the test space equal to *X*_*h*,*p*_, the SEM approximation of (3a)-(3b) consists in finding *ϕ*_*δ*_*i*,*e*__ ∈ *X*_*h*,*p*_ such that for all *ψ*_*i*,*e*_ ∈ *X*_*h*,*p*_ and for every *k*

(7a) χC∑k∂Vδ∂t,ψik+∑kaikϕiδ,ψi=−∑kχIion,ψik+∑kIstim,i,ψik,

(7b) −χC∑k∂Vδ∂t,ψek+∑kaekϕeδ,ψe=∑k+χIion,ψek+∑kIstim,e,ψek,

where *V*_*δ*_ = *ϕ*_*i*_*δ*__ − *ϕ*_*e*_*δ*__, while *a*_*i*,*e*_*k*__(*u*, *v*) = ∫_*Ω*_*k*__∇*u* ^*T*^ *σ*_*i*,*e*_∇*v* *dΩ*_*k*_ and (·, ·)_*k*_ denotes the inner product in *Ω*_*k*_. For the numerical realization of the SEM we resort to the so-called GNI approach (see, e.g., [16]), which consists in exploiting numerical integration for computing integrals. With this choice, the semidiscrete form of the bidomain equations becomes the following: find *ϕ*_*δ*_*i*,*e*__ ∈ *X*_*h*,*p*_ satisfying given initial and boundary conditions and such that for all *ψ*_*i*,*e*_ ∈ *X*_*h*,*p*_

(8a) χC∑k∂Vδ∂t,ψip,k+∑kaiϕiδ,ψip,k=−∑kχIion,ψip,k+∑kIstim,i,ψip,k,

(8b) −χC∑k∂Vδ∂t,ψep,k+∑kaeϕeδ,ψep,k=+∑kχIion,ψep,k+∑kIstim,e,ψep,k,

where the notation (·, ·)_*p*,*k*_ indicates that the integrals on *Ω*_*k*_ are computed using Gauss-Lobatto numerical integration formulas on the corresponding reference element $\hat{\Omega }$.

#### 3.5.1. Semidiscrete Form of the Bidomain Equation

In terms of the SEM basis, the spectral element solution is *ϕ*_*δ*_*i*,*e*__(**x**, *t*) = ∑_*l*=1_^*N*^Φ_*l*_^(*i*,*e*)^(*t*)*N*_*l*_(**x**) where Φ^(*i*,*e*)^ represent the unknown nodal value. If we denote by *M* = {*m*_*lm*_} the diagonal spectral element mass matrix and with *K*_*i*,*e*_ the symmetric intra and extracellular stiffness matrices; with elements

(9) $$\begin{array}{c} k_{lm}^{i,e}=\int_{\Omega }\sigma_{i,e}\nabla N_{l}\nabla N_{m}d\Omega \end{array}$$

we may write the semidiscrete form of the bidomain equations in compact matrix form as follows:

(10) χCM∂∂tΦiΦe+KΦiΦe=MIstim,i−MIstim,e−χMIionw,c,V−MIionw,c,V

or

(11) $$\begin{array}{c} \chi CM\frac{\partial }{\partial t}\left[\begin{array}{c} \Phi^{\left(i\right)}\\ \Phi^{\left(e\right)} \end{array}\right]+K\left[\begin{array}{c} \Phi^{\left(i\right)}\\ \Phi^{\left(e\right)} \end{array}\right]=\left[\begin{array}{c} F^{\left(i\right)}\\ F^{\left(e\right)} \end{array}\right], \end{array}$$

where the bidomain mass and stiffness matrices *ℳ* and *𝒜* are defined as

(12) M=M−M−MM,K=Ki00Ke.

In a similar, but easier way, one can show that the SEM formulation of the monodomain problem reads

(13) $$\begin{array}{c} \chi CM\frac{\partial V}{\partial t}+KV=MI_{\text{stim}}-\chi MI_{\text{ion}}\left(\;w,c,V\;\right)=F \end{array}$$

along with given cell model, boundary and initial conditions.

### 3.6. Semi-Implicit Time Discretization

We use a mixed time-marching scheme which is implicit for the intracellular concentration variables while it is explicit for **V** and **w**. This is motivated by the fact that the components *w*_1_, …, *w*_6_ of **w** in (1c) are fully decoupled and each depends only on itself and *V*, whereas the component *c*_1_ of **c** has complex, non-linear dependencies on *V*, on one another and on **w**, making an implicit time-step much more expensive to compute.

**Step 1 .**

Given the potential **V** ^*n*^ at the previous time *t*_*n*_, solve

(14) wn+1−ΔtRVn,wn+1=wn,cn+1=cn+ΔtSVn,wn+1,cn,

where Δ*t* denotes the time step size.

**Step 2 .**

Find Φ_*i*_^*n*+1^ and Φ_*e*_^*n*+1^ by solving the linear system

(15) $$\begin{array}{c} A\xi^{n+1}=b, \end{array}$$

where

(16) A=AtM+Ki−AtM−AtMAtM+Ke,ξn+1=Φin+1Φen+1,b=AtMVn−χMIionVn,wn+1,cn+1+MIstim,i−AtMVn+χMIionVn,wn+1,wn+1−MIstim,e

with *A*_*t*_ = *χC*_*m*_/Δ*t* and **V** ^*n*^ = Φ_*i*_^*n*^ − Φ_*e*_^*n*^. For the solution of (15) we exploit classical Krylov methods, taking into account the linear system consistency: see, for instance, [30, Theorem 2].

Clearly, the effective solution of (15) requires the choice of an optimal preconditioner, especially for the bidomain case. This is a difficult task which truly deserves a dedicated investigation. In particular, algebraic multigrid preconditioners (AMG) have been applied to the bidomain equations, often outperforming standard methods like the ILU or the block Jacobi preconditioner adopted in this work [31]. Quite often, AMG preconditioners are applied to the parabolic-elliptic formulation of the bidomain equations, while our SEM implementation stems from the coupled parabolic-parabolic problem (see Section 3.1). Analysis of AMG preconditioning for the SEM discretization of the coupled parabolic-parabolic problem (3a)-(3b) will be the object of a future paper, along with a study on the condition numbers of SEM matrices, both unpreconditioned and preconditioned with different methods.

## 4. Results and Discussion

In order to validate the proposed spectral element scheme we start analyzing its performance for monodomain and bidomain simulations using a well know benchmark on a simplified geometry. Then, to further assess the ability of the method to cope with the additional complexities associated with organ scale cardiac electrophysiology models we present the results of simulating monodomain activity on a realistic human heart geometry. Numerical experiments were performed at the CRS4 High Performance Computing centre comprising more than 3000 processors, grouped into 382 nodes of 8 Intel Xeon (2.8 GHz) cores, connected through a low latency Infiniband network.

### 4.1. The Monodomain Niederer Benchmark

**Figure 1:**
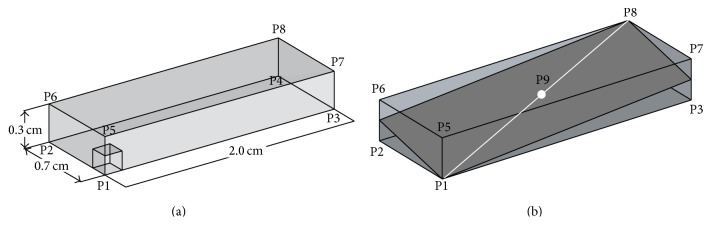
(a) The Niederer benchmark. (a) Domain dimensions with the 0.15 × 0.15 × 0.15 cm cubic stimulus region. (b) Activation times were evaluated at points P1 and P9 as well as along the line from P1 to P8. Plots of the activation along the plane shown are provided in two dimensions.

We use the benchmark proposed in [32]: it is a consensus cardiac tissue electrophysiology model problem that is used for evaluating and verifying cardiac tissue electrophysiology simulators. The problem geometry is defined as a parallelepipedal portion of cardiac tissue (*cuboid* or* slab*) with dimensions of 0.3 × 0.7 × 2.0 cm (see Figure 1), characterized by transversely isotropic conductivity with fibres aligned to the long axis of the computational domain.

**Figure 2:**
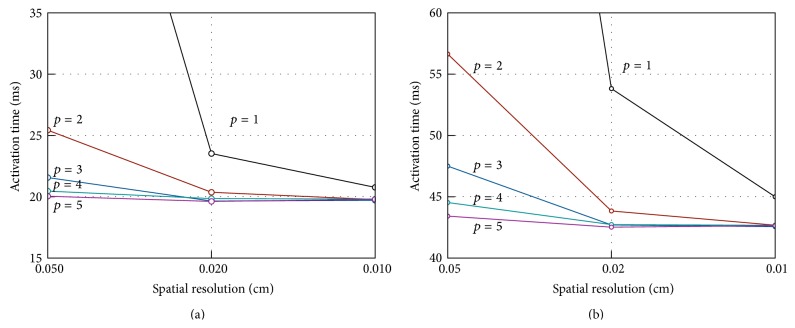
Activation times at cubic centroid P9 (a) and at point P8 (b) for monodomain solutions with varying spatial resolution and degree *p*.

Intralongitudinal, intratransversal, extralongitudinal, and extratransversal conductivities are assumed as 0.17, 0.019, 0.62, and 0.24 S/m. The stimulus current is applied to a cubic region of 0.15 × 0.15 × 0.15 cm located at one corner of the computational domain. The monodomain equations coupled with ten Tusscher and Panfilov model cell model [33] are solved for all simulations. We set a simple diagonal preconditioner, using a tolerance 10^−6^ for the Conjugate Gradient iterative algorithm. Activation times at the points shown in Figure 1 are evaluated at spatial resolution of 0.05, 0.02, and 0.01 cm and varying temporal discretisation for PDE (Δ*t* = 0.05, 0.01 and 0.005 ms). In the present case, activation time is defined as the unique time when the membrane potential passes through 0 mV upon first activation. In the following, we focus on the effect of varying the spectral degree *p* for the transmembrane potential *V*. Due to space constraints, we will present only data obtained using a temporal time step for PDE Δ*t* = 0.01 ms. Selected quantitative data for the simulations described above are presented in Table 1.  Figure 2(a) illustrates the measured activation times at the centroid with *p* = 1 to 5 for the three levels of spatial refinement, displaying the difference that high order methods brings. While inaccuracy is present with *p* = 1 for all spatial discretisations, we can see that *p* = 2 on the finest mesh and *p* = 3 on the intermediate mesh resolution are sufficient to get a practically converged solution. Note that using *p* = 4 and *p* = 5 we can mitigate the adverse coarsest mesh effects on the measured centroid activation times. In fact (see Table 1) the computed relative error with respect to the finest mesh solution with *p* = 5 is 3.398% and 1.286% for *p* = 4 and *p* = 5, respectively. The activation times at point P8 are showed in Figure 2(b) for varying degree *p* and different mesh resolutions. Due to error accumulation as the wave propagates across the computational domain, the effect of using higher degree *p* at lowest space resolution is slightly less favourable. The relative error with respect to the finest mesh solution with *p* = 5 is equal to 4.420% when *p* = 4 and 1.822% when *p* = 5. However, as the mesh is refined, the conduction velocity of the activation wave is again well represented with *p* = 2 and *p* = 3, on the finest and intermediate mesh resolution, respectively.

**Figure 3:**
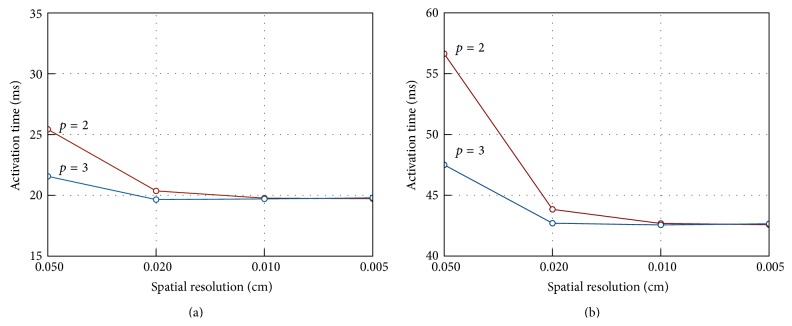
Activation times at slab centre P9 (a) and point P8 (b) for monodomain solution with *p* = 2 and *p* = 3 on meshes with *h* = 0.05 to *h* = 0.005 cm.

To test if these results were converged at these spatial scales we performed highly refined simulations with a 0.005 cm spatial resolution. Results for the cuboid centroid and for point P8 with *p* = 2 and *p* = 3 are illustrated in Figure 3. The degrees of freedom and the numbers of nonzero entries of the solution matrices (NNZ) for the various meshes in the simulations give some insight into the computational workload associated with the solution of the monodomain problem using spectral elements for a given degree of accuracy. In this case relative accuracy is measured by relative error on the activation time at a specific point in the computational domain with respect with a reference solution. In fact, we may consider that for a given cellular and electrophysiology model at a given spatial and temporal resolution the overall computational burden is essentially related to the one required for the iterative solution of the sparse linear system arising from PDEs discretization. Using iterative methods, computational effort is driven by matrix-vector multiplication whose cost, in turn, is essentially determined by NNZ, the number of nonzero entries of the solution matrix. In the case of spectral element method the latter are the most sensible parameter, as it is shown in Table 1 where it is apparent that they grow more than linearly for higher degrees *p*. If, for the moment, we disregard any optimisation issue of the solution of the linear system, we can assume NNZ as a rough estimate of the computational workload required to solve the problem for a given iterative algorithm and preconditioner. It is worth observing that assuming as a reference the solution generated using *h* = 0.01 cm and *p* = 5 these data show that a practically converged solution with *p* = 2 with *h* = 0.01 cm or *p* = 3 with *h* = 0.02 or 0.01 cm requires about 10% of the effort required for the reference solution in the worst case.

**Figure 4:**
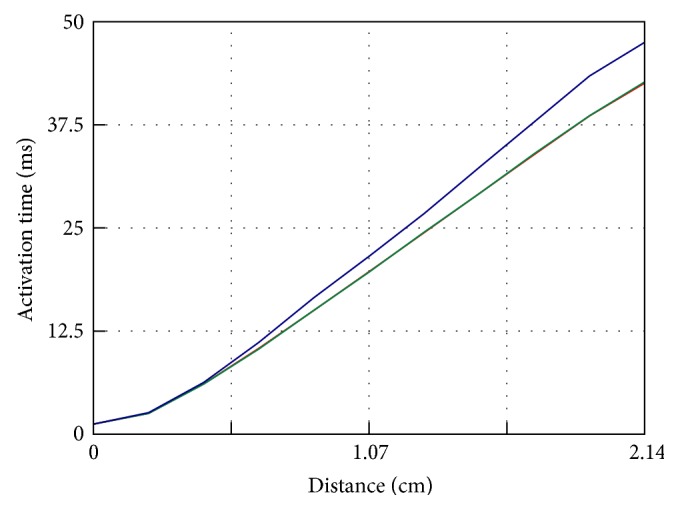
Activation times on the diagonal from P1 to P8 for *p* = 3 for monodomain solutions with Δ*x* = 0.01 cm (red line), 0.02 cm (green line), and 0.05 cm (blue line). Note that data for *h* = 0.01 cm and *h* = 0.02 cm are indistinguishable.

To show how errors are distributed in space, we computed the activation times on the diagonal from points P1 to P8 for *p* = 3 for varying mesh resolutions. Results are shown in Figure 4. The graph represents the activation times of points distributed along a line of roughly 21.4 mm in length. Ten points at equal intervals are selected along this line, and the activation time for each point is interpolated from the hexahedron which contains it. These curves highlight that for this choice of *p* there is essentially no dependence on spatial resolution except for the lowest one. All curves share a common morphology with the activation velocity slightly increasing as the mesh resolution improves. Note that in Figure 4 the solution with *h* = 0.02 cm (green line) cm is virtually not distinguishable from the solution with *h* = 0.01 cm (red line). Furthermore, boundary effects are not seen for intermediate to high spatial resolutions and remain limited for *h* = 0.05 cm.

**Figure 5:**
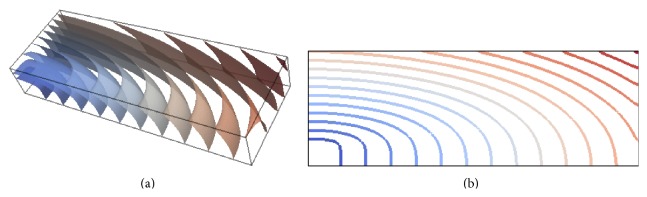
Isosurfaces of the monodomain activation times on the *h* = 0.02 cm mesh with *p* = 3. The activation times are represented by the colour map from dark blue (0 ms) to dark brown (45 ms) with contour bands at 3 ms intervals (a). Coloured contour map of the activation times on the plane depicted in Figure 1 for the same combination of *p* and *h* (b).

To determine the impact of conduction velocity error in directions perpendicular to the fibres due to the lower conductivity, we show in Figure 5 the isosurfaces of activation times on the *h* = 0.02 cm mesh with *p* = 3. For the sake of comparison with the data published in [32] we also provide the plots of the activation along the plane shown in Figure 1. Note that, for this choice of *p* and *h*, propagation along and across the preferential fibre direction does not affect activation wave curvature. These data also show the ability of the proposed method to capture off-fibre conduction velocities at relatively coarse spatial resolution and demonstrate visually that no boundary effects are present.

**Figure 6:**
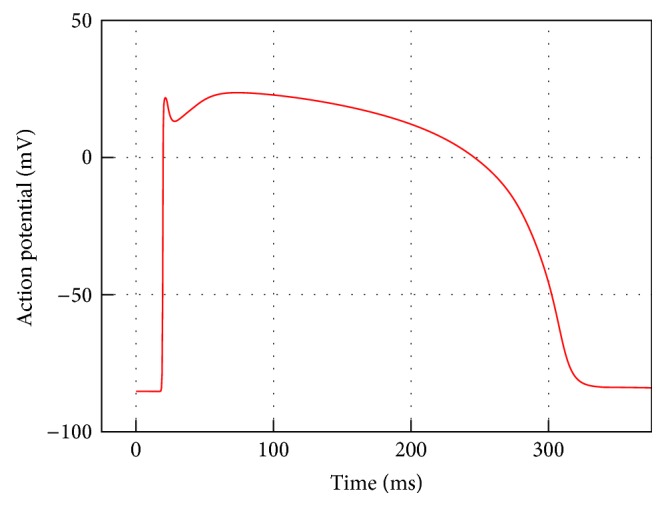
Monodomain action potential time history at point P9 along the cuboid diagonal for a 350 ms simulation.

To complete this test case, Figure 6 shows the activation potential time history at the cuboid centroid for a 350 ms simulation. It can be seen how for the transmembrane potential *V* depolarization, plateau and recovery phases are well reproduced.

### 4.2. The Bidomain Niederer Benchmark

We studied bidomain simulation with the same benchmark used in Section 4.1 for the monodomain case. As before, we consider a simulation domain of size 0.3 × 0.7 × 2.0 cm at mesh discretisations of 0.05, 0.02, and 0.01 cm and anisotropic conductivity. The domain was presented in Figure 1 and further details were given in Section 4.1.

**Figure 7:**
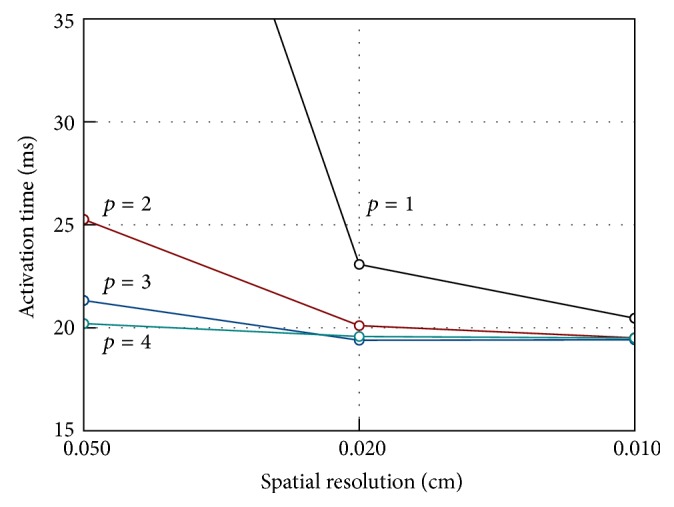
Activation times at cubic centroid P9 for bidomain solutions with varying spatial resolution and degree *p*.

The measured activation times at the cuboid centroid are shown for a range of spectral degrees *p* and varying spatial resolutions in Figure 7. Note how cubic and quartic degrees display superior accuracy to lower degrees by the second coarsest level. Furthermore, on the finest mesh resolution, the solution with *p* = 2 is undistinguishable from the other solutions showing that with *h* = 0.01 cm roughly equivalent accuracy can be attained by *p* = 2.

**Figure 8:**
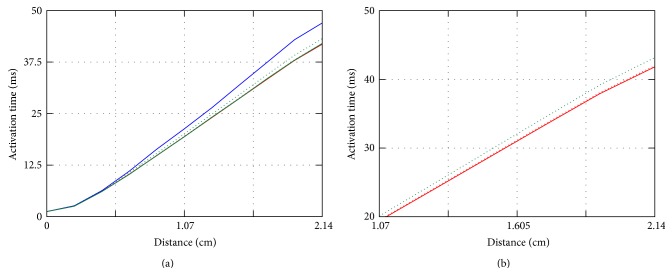
(a) Activation times on the cuboid diagonal for bidomain solutions with degrees *p* = 3 (continuous line) and mesh resolution *h* = 0.01 cm (red), 0.02 cm (green), and 0.05 cm (blue) and *p* = 2 (dotted line). Solutions for *p* = 2 are shown only for the two finest mesh resolutions. (b) Details of (a) solution are shown for *p* = 2 and *p* = 3 for *h* = 0.01 cm (red), 0.02 cm (green).

These data suggest that *p* = 3 on the second coarsest mesh or alternatively *p* = 2 on the finest spatial resolution is practical *p* and *h* combinations also for the bidomain model. This is further illustrated from the results of computing activation times along the cuboid diagonal.  Figure 8 shows how errors are distributed in space for selected combinations of *p* and *h*. Essentially, at the suggested mesh discretisation the solutions for *p* = 2 and *p* = 3 are practically undistinguishable. Furthermore, in all cases, the boundary effects are minimal. Note that the effect of using *p* = 2 on the second coarsest mesh reduces to slightly underestimate the conduction velocity with no apparent effects on the morphology of the resulting curve. Data for point P8, not shown here due to space constraints, confirm the findings the monodomain case we discussed in Section 4.1.

**Figure 9:**
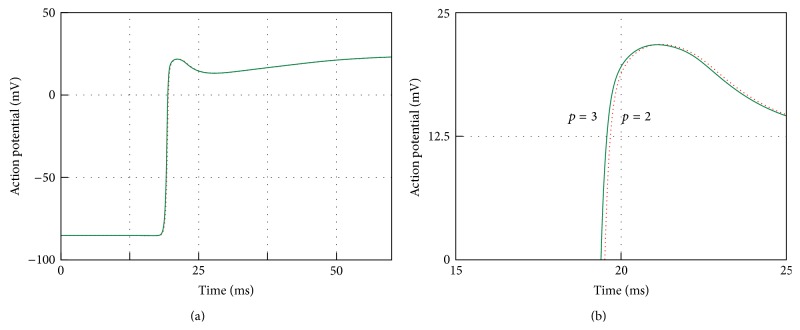
The bidomain action potential at the slab centre (point P9) for *p* = 2 (dotted line) and *p* = 3 (continuos line) and with mesh mesh resolution 0.01 cm (red line), 0.02 cm (green line). The action potentials for the full duration of the simulation are shown on the left. On the right the differences between *p* = 2 and *p* = 3 are shown by zooming in on the AP upstroke (15–25 ms).

Further investigations on the shape of the wave produced with the simulations quantify the differences one might expect in using one or the other combination of *p* and *h*. This is visually illustrated in Figure 9 which shows the action potential time history at the centroid of the cuboid diagonal for a 60 ms simulation. Results shown in the right panel confirm that with *p* = 2 there is still a minimal overestimate of the conduction velocity with no practical effects when compared with the converged solution with *p* = 3.

These data show that *p* = 2 on the finer mesh or *p* = 3 on the second coarsest mesh ought to be preferred over any other combination of *p* and *h* given that this produces equivalent accuracy. However, details on the degrees of freedom and on the number of nonzero entries of the solution matrix, 6,898,002 and 258,371,604 for *p* = 2 and 2,935,352 and 178,361,104 for *p* = 3, indicate that this latter combination produces a fully converged solution at roughly 69% of the effort required to solve the problem in the former case.

**Figure 10:**
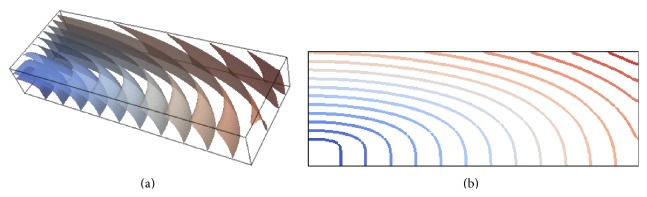
Isosurfaces of activation times on the *h* = 0.02 cm mesh with *p* = 3 for the bidomain solution. The activation times are represented by the colour map from dark blue (0 ms) to dark brown (45 ms) with contour bands at 3 ms intervals (a). Coloured contour map of the bidomain activation times on the plane depicted in Figure 1 for the same combination of *p* and *h* (b).

Finally, Figure 10 confirms the ability of the proposed method to capture off-fibre lower conduction velocities when solving the bidomain problem. Note how the wave activation pattern propagates for all times with no relevant boundary effects.

### 4.3. High-Resolution Anatomically Realistic Heart

**Figure 11:**
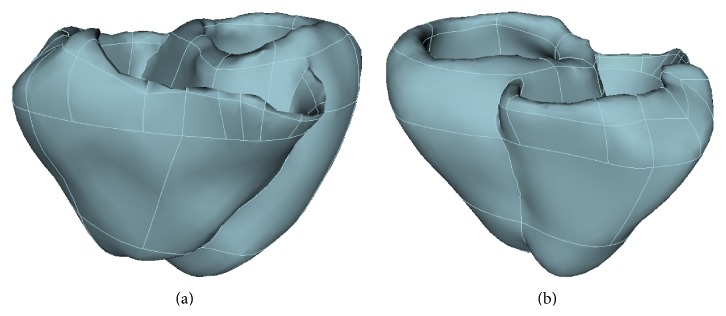
Full scale three-dimensional geometrical model of a human heart generated from a ventricular surface definition obtained from computed tomography (CT) images. Panels (a) and (b) show anterior and posterior views of the model. White lines indicate the subvolume partition used for unstructured all-hexahedra mesh generation.

We continue the assessment of the method presenting the results of simulating monodomain activity on an anatomically realistic, high-resolution cardiac computational mesh. At present, fully automatic all-hex mesh generation of complex solids, such as a whole heart, is not yet possible, and most volumes require some measure of decomposition before they can be meshed with a hexahedral meshing scheme. To tackle this problem, we developed a computational pipeline based upon CUBIT (Sandia Laboratory, http://cubit.sandia.gov), an advanced and robust solid modeler and 3D unstructured hexahedral mesh generator that offers state-of-the-art capabilities to design, assess, and improve the quality of a mesh in terms of both geometrical and numerical accuracy. Starting from a ventricular surface obtained from CT images and represented by means of triangular elements, a volume model is generated from which a decomposition into automatically meshable subvolumes can be constructed. A conforming unstructured all-hexahedra mesh is then semiautomatically generated and quality checks and mesh validation are carried out on each subvolume. At the end of the process the quality of resulting mesh with respect to standard finite element metrics is assessed and the mesh is finally validated for use in simulation studies. The surface definition we used was derived from the statistical shape model constructed from a training set comprising 100 asymptomatic and pathologic subjects described in [34]. Data were originally provided by the CISTIB at the Universitat Pompeu Fabra, Spain.  Figure 11 shows an anterior and a posterior view of the complete human heart model after subvolume partition.

**Figure 12:**
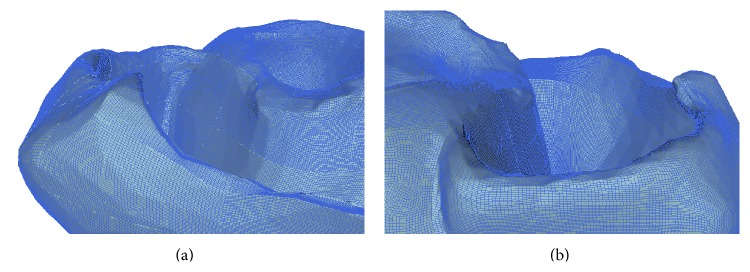
Full scale complete human heart mesh derived from CT images. Panels (a) and (b) show two enlarged views of the high-resolution unstructured all-hexahedral mesh used to solve the monodomain equations.

Using the pipeline outlined above we are currently able to generate whole human heart meshes with very fine average edge length equal to 0.018 cm (35,021,521 nodes and 34,269,632 elements). Due to hardware constraints the version of the mesh used in the simulation study presented below has an average edge length of 0.036 cm and consists of 4,471,781 nodes and 4,283,704 elements.  Figure 12 shows some detailed views of the all-hexahedral mesh we used in our simulations. In the present benchmark the cellular membrane dynamics were defined by the phase-I Luo-Rudy cell model [24], although any other ionic model could have been used. Spatial variation in fibre direction is not accounted for and homogenous membrane properties are assumed throughout all volume. The isotropic baseline conductivities were *σ*_*i*_ = diag(0.175,0.175,0.175) S/m and *σ*_*e*_ = diag(0.7,0.7,0.7) S/m, where diag(*x*, *y*, *z*) is a 3 × 3 diagonal matrix with values *x*, *y*, *z* along the diagonal. Other parameters were *χ* = 1400 cm^−1^ and *C* = 1.0 *μ*F/cm^2^. A stimulus of magnitude 4.0 × 10^3^ *μ*A/cm^2^ and duration of 2.0 ms was applied to surface nodes within the apical region of the mesh (*z* < 5.0 cm) which elicited the propagation of a quasi-planar wavefront in approximately an apicobasal direction.

**Figure 13:**
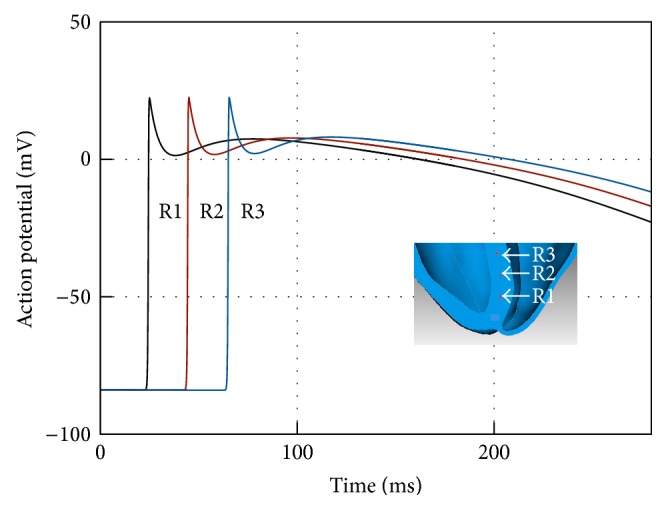
Activation potential time history for a 250 ms human heart bidomain simulation at various points located at human heart internal septum. The insert shows the location of the points where the time history is registered.

**Figure 14:**
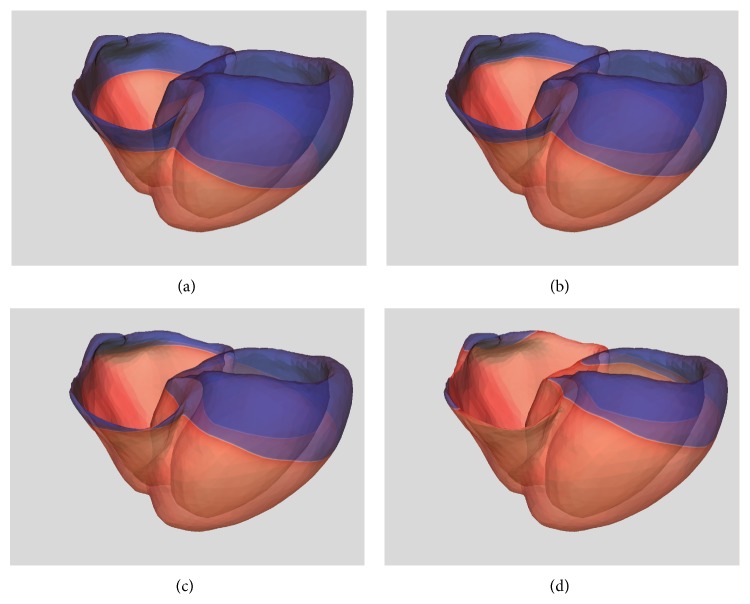
Spectral element approximation (*p* = 3) of the transmembrane potential at (a) *t* = 75 ms, (b) *t* = 85 ms, (c) *t* = 95 ms, and (d) *t* = 105 ms on a realistic heart geometry. Potential distribution ranges from blue (resting value) to red (depolarization). Transparency of the geometrical model exposes epicardial and endocardial activity.

**Figure 15:**
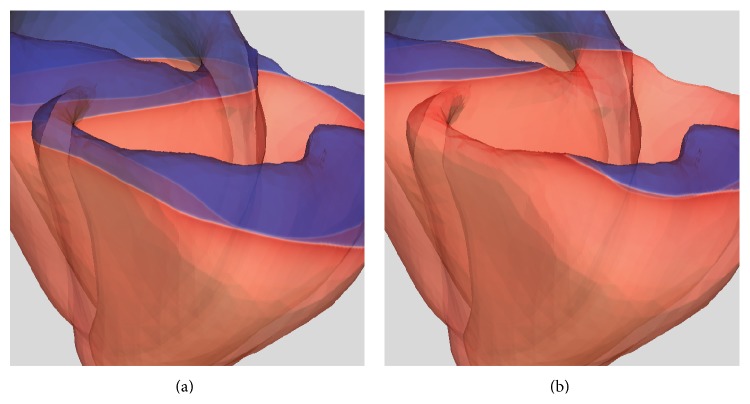
Enlarged snapshot of the transmembrane potential at (a) *t* = 95 ms and (b) *t* = 105 ms. Here we focus on resolution of the activation wavefront in organ scale complex electrophysiology computational models.

Figures 13–15 show the results of simulating 250 ms of monodomain activity on the heart geometry described above. Simulations were run with spectral degree *p* = 3 and time steps of 0.005 ms and 0.01 ms were used for the ODE and PDE solves, respectively. The simulations were run on 128 cores. Our FORTRAN90/MPI parallel spectral element code is based on the parallel library PETSc from the Argonne National Laboratory (http://goo.gl/LGvZKZ) and embeds METIS for partitioning spectral element meshes (http://glaros.dtc.umn.edu/gkhome/views/metis). The iterative linear solver used was Conjugate Gradient with an ILU preconditioner. The total number of degrees of freedom of the problems is equal to 117,351,445 with 7,097,533,909 nonzero matrix entries.

We do not have a reference solution in this case, but we should expect to achieve a degree of accuracy similar to the *h* = 0.02 cm or, in the worst case, to the *h* = 0.05 cm Niederer benchmark with *p* = 3. Note that the quality of numerical simulations can be improved at runtime simply by increasing the polynomial degree of the basis functions used in the numerical scheme without the need of generating a new mesh as it would be the case for standard finite elements.  Figure 13 shows the action potential time history for three points located in the heart internal septum. These plots demonstrate the ability of the proposed method of capturing the shape of a travelling steep front at this spatial resolution. The absence of oscillations at the end of the depolarization phase further corroborates this observation.

Panels from *t* = 85 ms to *t* = 105 ms in Figure 14 show the resulting electrical activation sequence of an action potential cycle. Note how the wavefront is well represented and propagates without distortion at this mesh resolution. Convergence studies using finite elements as a comparison, discussed below, demonstrate that this is definitively not attainable at the present spatial resolution with standard low order methods. Finally, to better appreciate the quality of the wavefront resolution some zoomed snapshots of the transmembrane potential at 95 ms and 105 ms after activation are presented in Figure 15.

The analysis of parallel performance of the SEM algorithm deserves a dedicated study and is not included in this work. As a matter of fact, SEM bring overall matrices which are more dense and smaller in size with respect to low order methods: this, in principle, may allow good computational efficiency. Without going into details, this depends on (i) the way matrices are split between parallel tasks, (ii) the low number of interface nodes (i.e., nodes shared by different tasks) typical of SEM, as they need less grid points to provide the same accuracy, and (iii) the fact that parallel efficiency roughly depends on the ratio between computational effort of each task and amount of message passing. Improvements in parallel efficiency for increasing value of the spectral degree have been measured in other fields of application long time ago; see for example, [35]. Last, the choice of the preconditioner may significantly affect the parallel performance.

### 4.4. Computational Effort: A Comparison with Finite Elements

We exploit the previous example to assess the accuracy and computational cost of our code in comparison with standard finite elements. Because of the time-consuming nature of performing such investigation across a range of different parameters in three dimensions, we used a very small test domain. To this scope, we extracted from the right ventricle wall of the human heart geometry described above a rectangular cuboid spanning from to epicardial to endocardial wall surface with a volume of approximately 0.365 cm^3^. We then solved an isotropic monodomain problem on this subvolume, using linear finite elements on a series of progressively uniformly refined computational meshes. The meshes we used have an average spatial resolution of *h* approximately equal to 0.02, 0.01, 0.005, and 0.0025 cm.

**Figure 16:**
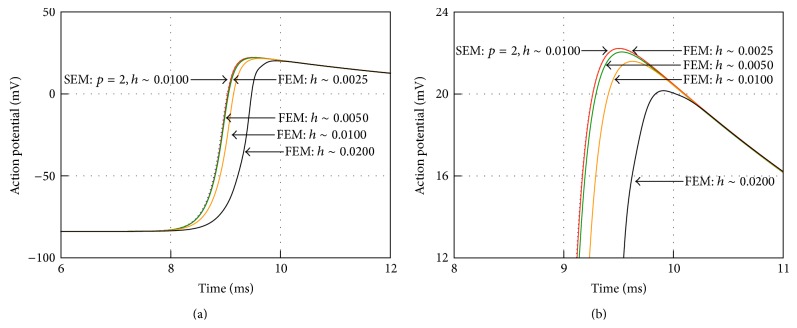
Space-refinement convergence experiments for monodomain action potential in the human heart subvolume. Continuos lines indicate FEM solution with mesh resolution 0.02 cm (black), 0.01 cm (orange), 0.005 cm (green), and 0.0025 cm (red). Dotted line denote SEM solutions with *p* = 2 and spatial resolution 0.01 cm (blue). The action potentials for the full duration of the simulation are shown on the left. On the right the AP upstroke (8–11 ms) is shown for the same range of mesh refinements.

The parameters used for the simulation were the same used for the heart-scale problem. A stimulus of magnitude 4.0 × 10^3^ *μ*A/cm^2^ and duration of 2.0 ms was applied to the bottom surface nodes of the cuboid. The resulting time histories at a receiver located in the external surface of the subvolume at a distance from the stimulation face approximately equal to 80% of its maximum vertical dimension are shown in Figure 16 where they are compared with a SEM solution obtained with *p* = 2 and average *h* approximately equal to 0.01 cm.

These curves highlight that finite elements progressively converge to the SEM solution as the mesh resolution improves. In particular the FEM solution with *h* = 0.0025 cm (continuous red line) is virtually not distinguishable from the solution obtained with SEM using *p* = 2 and *h* = 0.01 cm (dotted blue line).

Table 2 shows a comparison between FEM and SEM analyses in terms of DOFs, nonzero entries, and CPU-time to time-advance the monodomain equation. The SEM are able to provide the same accuracy of the FEM solver using approximately 20% of DOFs: this is not too far from our findings in different fields of applications. The DOFs ratio drives quite well the CPU-time for time-advancing the ODE system associated with the cell model (28%). While the SEM NNZ are roughly half of their FEM counterpart, the CPU-time for time-marching the monodomain equation with SEM is about 1/3 of that required by FEM. There are several reasons for such deviation, including the number of iterations needed to solve the linear system at each time-step (slightly in favour of SEM), the additional cost of the preconditioner when solving the linear system (15), the fact that in modern computer architectures memory access and data movement are at least as expensive as number crunching.

These results are, in our opinion, quite promising; we understand that the potential of method we are presenting to reduce the amount of time required to perform large scale electrophysiology simulations has to be further confirmed with a thorough comparison of the spectral element and the finite element methods on both the monodomain and bidomain equations. This will be the subject of a forthcoming contribution.

## 5. Conclusions

In this paper we presented an effective high order discretisation technique based on the spectral element method for the study of electrophysiological wave propagation. A fully unstructured all-hexahedra approach implementation of the method has been illustrated and applied to the solution of full 3D monodomain and bidomain models. A careful validation of the implementation was performed on a consensus monodomain benchmark with anisotropic conductivities which was also extended to the bidomain case. At the organ level, a full scale simulation model of the heart was used to assess the ability of the proposed method to cope with the additional complexities associated with large scale cardiac electrophysiology models. As for our knowledge, few attempts have been done to exploit spectral elements in electrocardiology; our work represents an extension to these studies, which were typically treating with over-simplified computational domains. The approach here proposed enjoys many advantages over standard techniques, such as considerably higher convergence rates, geometrical flexibility, and, in our experience, the possibility of using elements with large aspect ratios without significant deterioration in accuracy. Furthermore, the flexibility and robustness of the method, as demonstrated in solving complex three-dimensional problems at organ scale, are quite appealing and promising and will be further explored in combination with the development of advanced approaches to maximise code performance. We are aware that there are a number of aspects that deserve further investigation. For example we have not covered the experimental evaluation of the parallel performance of the method at hand. This will be subject of a forthcoming contribution. Further improvements of this work may include the exploitation of new hardware devices like GPGPUs as well as the investigation of efficient and scalable preconditioners that would allow an increased performance.

## Figures and Tables

**Table 1:** Monodomain activation times at the corner P8 and the centroid P9 on the domain shown in Figure 1 using various *h* and *p* values. Percentage relative errors on activation times computed assuming as a reference the solution provided by *h* = 0.01 cm and *p* = 5. Details on the degrees of freedom (DOF) and the numbers of nonzero entries of the solution matrices (NNZ) for the various meshes used in the simulations; percentage of DOFs and NNZs are computed with respect to those of the reference solution.

*h*	*p*	P8	P9	% error @ P8	% error @ P9	Elements	DOF	NNZ	% DOF	% NNZ
0.05	4	44,53	20,46	4.420	3.398	3,360	229,425	20,079,265	0.84	0.30
	5	43,42	20,04	1.822	1.286	3,360	442,401	54,425,001	1.62	0.81

0.02	2	43,84	20,36	2.798	2.916	52,500	442,401	15,894,801	1.62	0.24
	3	42,70	19,65	0.138	−0.685	52,500	1.467,676	87,712,876	5.39	1.31
	4	42,73	19,85	0.209	0.314	52,500	3,449,001	308,946,001	12.66	4.61
	5	42,52	19,62	−0.285	−0.846	52,500	6,701,376	840,210,876	24.61	12.55

0.01	2	42,68	19,77	0.085	−0.072	420,000	3,449,001	125,736,801	12.66	1.88
	3	42,56	19,71	−0.204	−0.409	420,000	11,539,801	696,717,001	42.37	10.41
	4	42,67	19,81	0.056	0.126	420,000	27,234,801	2,147,483,648	51.33	32.08
	5	42,64	19,79	0.000	0.000	420,000	53,054,001	6,694,583,001	100.00	100.00

**Table 2:** Details on the degrees of freedom (DOF) and the numbers of nonzero entries of the solution matrices (NNZ) for the human heart subvolume benchmark. Details for CPU-times for PDEs, ODEs, and PDEs + ODEs. Percentages are computed with respect to those of the FEM solution.

Method	*h* (cm)	*p*	DOF	NNZ	Mean PCG	CPU-time	CPU-time	Total CPU-time
					iterations	PDEs (s)	ODEs (s)	(PDEs + ODEs) (s)
SEM	0.0100	2	1,215,825	44,221,761	9.0	2.57	2.62	5.19
FEM	0.0025	—	6,290,417	93,516,241	10.2	7.19	9.12	16.31

SEM/FEM	400%	—	19.3%	47.4%	88.5%	35.7%	28.7%	31.8%
